# Could periodontitis be associated with overall and cancer-specific mortality risk; a 35-year Swedish cohort study

**DOI:** 10.2340/aos.v84.44915

**Published:** 2025-12-15

**Authors:** Freja Frankenhaeuser, Esa Korpi, Birgitta Söder, Håkan Källmén, Jukka H. Meurman

**Affiliations:** a Department of Oral and Maxillofacial Diseases, University of Helsinki, Helsinki, Finland; b Department of Pharmacology, University of Helsinki, Helsinki, Finland; c Department of Dental Medicine, Karolinska Institutet, Stockholm, Sweden; d Department of Psychology, Uppsala University, Uppsala, Sweden

**Keywords:** Periodontitis, mortality, medications, tooth loss, oral health

## Abstract

**Objective:**

Many studies have shown a link between inflammation and increased morbidity and mortality. We evaluated the association between baseline periodontitis, medication purchases, and mortality in a 35-year follow-up.

**Materials and methods:**

The sample and data are from a long-term follow-up of a random sample, from the greater region of Stockholm, Sweden, 1985 to 2017. The sample comprised 1,643 participants, initially clinically examined in 1985, and found to either have (*n* = 286) or not have (*n* = 1,357) periodontitis. Swedish national population and patient registers from 1985 to 2017 were used for analyses. Descriptive statistics, Chi^2^-test, Kaplan-Meier and Coxs proportional hazard regression models were used in the analyses. The outcome variable in the Kaplan-Meier and Cox’s regression analyses was time to death.

**Results:**

Periodontitis at baseline showed a positive association with all-cause and, particularly, with cancer-specific mortality and a poorer survival distribution. In addition, having lost two or more teeth at the age of 30–40 years, a higher dental calculus or gingival index at baseline, were identified as risk factors for overall mortality among the participants. In addition, alimentary tract and metabolism medications, systemic hormones, and antineoplastic and immunomodulating agents were associated with higher mortality risk.

**Conclusion:**

This study found that periodontitis diagnosed 35 years earlier was associated with an earlier death when compared with the then periodontally healthy participants.

## Introduction

Periodontitis is a chronic inflammatory disease of the gums with a complex interaction between the host’s immune reaction and bacteria, affecting 1 billion people globally [1]. It is characterised by periodontal pockets and loosening of the supporting structures of the teeth. The associated microbial colonisation and subsequent inflammation are mainly a result of poor oral health. Other risk factors include smoking, genetics, stress and systemic conditions. It has increasingly been linked with several systemic diseases including cardiovascular diseases (CVD), diabetes, cancers, respiratory diseases, and rheumatoid arthritis [2–7]. In our previous study, we observed that periodontitis was significantly associated even with all kinds of cancer [8]. However, despite this growing body of evidence, the potential influence of periodontitis on overall mortality risk and premature deaths remains underexplored.

Premature death refers to death that occurs before the average life expectancy within a given population. In 2022, the average life expectancy of women globally was 73.8 years, and of men 68.4 years [9]. This cohort study is based in the greater Stockholm region, Sweden. The Swedish life expectancy globally of women was 84.3 years and of men 81.0 years [9]. Life expectancy is on the rise. If comparing life expectancy in Sweden between the years 1970 to 2023, an increase from just over 77 years to nearly 85 years for women and from just over 72 years to nearly 82 years for men has been observed [10]. The main causes of death in Sweden in 2019 were ischemic heart disease, Alzheimer’s disease, stroke, chronic obstructive pulmonary disease (COPD), tracheal, bronchial, and lung cancers [11]. Cardiovascular diseases and cancer are the leading causes of premature death in 127 countries, and cancer is expected to surpass CVD as the leading premature cause of death globally within this century [12].

Previous studies have highlighted associations between poor oral health and tooth loss, and an increased risk for overall mortality and, particularly, mortality due to CVD, cancer, and respiratory diseases [13–16]. Furthermore, low oral health-related quality of life (OHQOL) has been associated with an increased risk of mortality [17].

Earlier research found an association between polypharmacy and mortality [18, 19]. Polypharmacy, the usage of five or more medications simultaneously, has increased worldwide. For example, in the United States of America (US) it was predicted that prescription medication purchases would increase between 10 and 12% in 2024 [20]. Furthermore, it has been proposed that prescription drug purchase rates could be used as a proxy for disease prevalence [21]. Still, the role of medication use, particularly as a reflection of systemic disease and comorbidity burden, is seldom examined in the context of oral health and mortality outcomes.

The aim of the present study was to investigate the influence of long-term periodontitis, tooth loss, and poor oral health on the time to death, by incorporating data on medication purchases. This research paradigm was on the potential pathways that link chronic inflammation to systemic health outcomes. The hypothesis is that periodontitis and poor oral health at baseline correlate positively with increased mortality. Our study cohort (*n* = 1,598) has been followed up since 1985.

## Means and methods

### Participants of the cohort

**Figure 1:**
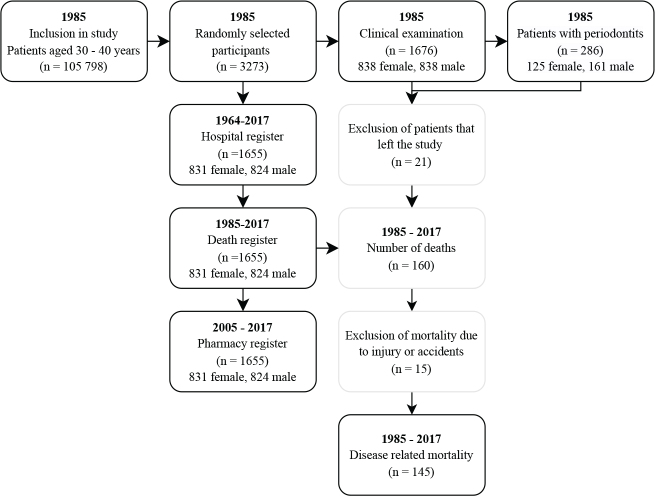
Evolution of data. Since the Prescribed Drug register started in 2005, the pharmacological data is missing from the years 1985–2004. To account for this the study was split into two and the analyses were done parallel to each other. The first data set included all deaths from 1985 to 2017 living participants (*n* = 1,495) and dead participants (*n* = 145). The second set included only the living (*n* = 1,495) and deceased participants (*n* = 103) between 2005 and 2017.

At baseline in 1985, a total of 1,676 participants underwent a clinical examination. These participants were selected from 3,273 randomised individuals, enrolled out of the basic cohort size of 105,798 from Stockholm metropolitan area, Sweden [22]. The subjects were all born on the 20^th^ of each month between the years 1945–1954. After a drop-out of 21 participants, the cohort size was 1655 subjects. In the analyses, 15 participants were further excluded, because their cause of death was due to trauma or accidents (*n* = 15). Finally, 1,640 participants were the material of the present study. Figure 1 shows the evolution of the subject material, and the registers used in the study. These registers are maintained by the National Board of Health and Welfare (Swedish: *Socialstyrelsen*) Sweden.

### Ethics approval

The Ethics Committee of the Karolinska University Hospital at Huddinge has approved the study (Dnr 2007/1669–31; 2012/590–32; 2017/2204–32). There was no patient involvement in the study as this was a register study with no direct patient involvement. The authors have obtained approval of informed consent from the participants at the beginning of the study.

### Causes of death

The National Cause of Death Register provides the official causes of death in Sweden. The register contains multiple variables but includes the principal cause of death, time of death, and whether alcohol, substances, or narcotics were present at the time of death. In the register, the causes of death are recorded by the World Health Organization (WHO) International Classification of Diseases versions ICD-9 (1979–1998) and ICD-10 (1999–2017), respectively.

### Hospital diagnoses before the year 1985

To assess the participants’ hospital diagnoses before 1985 and causes of death, ICD-8, ICD-9, and ICD-10 codes were used. Trauma and childbirth-related deaths were however, excluded. Correspondingly to the all-causes of deaths in the whole material, ICD-8 codes from the years 1968 to 1978 and ICD-9 codes from the years 1979 to 1998 were used.

### Medication purchases

Swedish National Prescribed Drug register spans from the years 2005 to 2017, and includes all the medication purchased by the participants of the cohort. In the register, the medications are categorised based on the Anatomical Therapeutic Chemical (ATC) classification system, organised into main groups (the first anatomical level of ATC), and subgroups (the second level of ATC). The ATC system consists of 14 main groups, of which 13 were included in this study. The ‘Various’ group (ATC code V) was excluded due to its heterogeneity and the limited number of recorded purchases. For each medication main group, participants were assigned coded as: 1 = purchased the medication and 0 = did not. The median of all medication purchases was calculated and values higher than the median was coded as 1 and below or equal to the median as 0.

### Oral health records of the participants

During the baseline clinical oral examinations in 1985, the participants underwent assessments that included counting the number of remaining teeth (third molars were excluded), the calculus index (CI), the plaque index (PI), and a modified gingival index (GI). The CI and PI were based on the simplified oral hygiene index (OHI-S). The modified GI was based on bleeding on probing (BOP) as well as the gingival appearance after probing on four sites of the tooth [22]. A median for each variable was calculated, and values higher than the median was coded as 1 and below or equal to the median as 0 for all indexes. The reason for missing teeth during the decades of follow-up had not been recorded in this database. Other factors recorded were halitosis, partial dentures, and gingival recession. Periodontal pocket probing depths (PD) were also measured. The PD was determined to be the nearest millimetre at six sites of the tooth. However, periodontal pockets greater than 5 mm were also recorded. A periodontitis diagnosis was set for participants who had 3 or ≥ 5 mm PD values in three different non-adjacent teeth [22].

### Tobacco usage

The participants’ use of tobacco products, including smoking and the use of Swedish snus, was recorded at baseline. Earlier tobacco habits were also recorded for the then non-smokers and ex-smokers. A variable for tobacco consumption was constructed. Being a smoker or snus user was coded as 1 and a non-user as 0 for the analyses. This was due to a major overlap in using both snus and being an active smoker.

### Socioeconomic status

At baseline in 1985, the participants were classified into socioeconomic groups based on income and educational attainment. Individuals with lower educational levels and lower or no income were categorised as having a lower socioeconomic status (coded as 1), while those with higher educational levels and greater income were categorised as having a higher socioeconomic status (coded as 0).

### Age

Age was recorded in years at baseline, time of death, and at cut-off in 2017. For alive participants their age at cut-off and for participants deceased between the years 1985 and 2017 their death age, were used in analyses adjusted for age.

### Statistical analyses

Data reorganisation, summation of the registers, and calculations were performed using Visual Studio Code 2 and Python 3.9.10 (64-bit). Descriptive statistics involved comparing alive and deceased participants and their baseline characteristics using frequencies and Chi^2^-tests, with a significance level set at 0.05.

To adjust for medication purchases, and subsequently, comorbidities, the data and analyses were split up and analysed as two different groups. Since the Swedish National Pharmacology Register is newer than our study originally, we analysed separately the deaths occurring only between the years 2005 and 2017 and all deaths occurring between the years 1985 and 2017. This allowed us to include the medication purchases in the analyses, while simultaneously being able to compare the results to those from the total material.

To compare survival functions for the participants with periodontitis at baseline and the periodontally healthy participants, Kaplan-Meier-tests were performed. To statistically compare the survival between the groups, log-rank-test (Mantel-Cox), Breslow (Generalized Wilcoxon), and Tarone-Ware were all used. The significance value was set at α = 0.05.

Cox’s proportional hazards regressions were used to assess the association between different independent factors and the time to death. The hazard ratio (HR) indicates the degree to which the variable affects the risk of death. The use of Cox’s proportional hazard regression models was to assess the association between survival time of the participants and different covariates, to minimise bias. To address the potential confounding, the regression analyses were split into three different models. The first model only incorporated mortality and oral health factors, the second model also adjusted for covariates such as gender, age, tobacco consumption, baseline socioeconomic status in 1985, and the presence of hospital diagnoses before 1985 (excluding trauma and childbirth). Model 3 was adjusted for all covariates in model 2 and, in addition, the medication purchases among the participants. The different main groups of the medications purchased were coded as ‘1’ if purchased and ‘0’ if not purchased. To study the role of polypharmacy, the analyses in the model 3 were separately accounted for the total number of medication purchases above the median (1) and below or at the median (0). Since the pharmacological register started in 2005, the pharmacological data are missing from the years between 1985 and 2004. Hence, model 3 is not included in Table 3. The statistical analyses were conducted using Statistical Package for the Social Sciences (SPSS) 28.0 software.

## Results

The basic characteristics of the cohort and the number of deaths are presented in Table 1. In 2017, the mean age for the participants was 67.6 years (standard deviation [SD] = 2.83). The mean age of death between the years 1985 and 2017 was 58.4 years (SD = 8.08) in the whole material. For the participants who had been diagnosed with periodontitis at baseline, the mean age of death was 60.7 years (SD = 7.15). Altogether 91.2% of the cohort was alive in 2017, meaning that 8.8% were deceased. Among the participants with periodontitis at baseline, 13.8% were deceased.

**Table 1:** Basic characteristics of the cohort as analysed between the years from 1985 to 2017 and, respectively, between the years from 2005 to 2017.

	1985–2017		2005–2017	
	Alive	Dead	Alive	Dead
Participants	1495	145	1495	97
Age (Mean)	67.6 SD: 2.8	58.6 SD: 7.9	67.6 SD: 2.8	62.8 SD: 4.4
Age (Median)	68.0	60.0	68.0	63.0
Men	730 (48.8%)	83 (57.2%)	730 (48.8%)	52 (53.6%)
Women	765 (51.2%)	62 (42.8%)	765 (51.2%)	45 (46.4%)
Tobacco use at baseline	518 (34.6%)	76 (52.4%)	518 (34.6%)	52 (53.6%)
Hospital diagnosis before 1985	519 (34.7%)	59 (40.7%)	519 (34.7%)	36 (37.1%)
Low socioeconomic status at baseline	292 (19.5%)	41 (28.3%)	292 (19.5%)	20 (20.6%)
Periodontitis at baseline	243 (16.3%)	39 (26.9%)	243 (16.3%)	27 (27.8%)
Lost two or more teeth in 1985	400 (26.7%)	57 (39.3%)	400 (26.7%)	33 (34.0%)
PI	598 (40.0%)	74 (51.0%)	598 (40.0%)	46 (47.4%)
CI	704 (47.1%)	90 (62.1%)	704 (47.1%)	59 (60.8%)
GI	721 (48.2%)	93 (64.1%)	721 (48.2%)	58 (59.8%)

PI: High plaque index; CI: High calculus index; GI: High modified gingival index.

These data show the distribution of data among the sample. The data set is split up into two periods: 1985–2017 and 2005–2017. The analyses were performed in parallel for both groups. The data from 1985–2017 and 2005–2017 have been analysed separately but are shown next to each other. The percentages are calculated for each column. The age is given in years. Different oral health variables recorded clinically at baseline in 1985: Periodontitis at baseline. The loss of two or more teeth at baseline.

**Figure 2:**
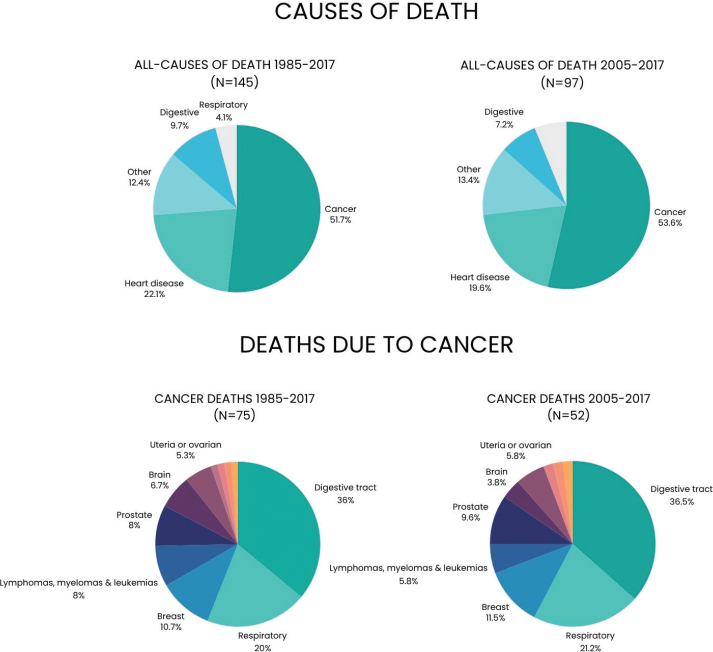
The figure depicts all causes of death in the study and then cancer-specific causes of death during the years 1985–2017 compared to the years 2005–2017.

From the years 1985–2017, a total of 145 participants died of medical causes. More details are shown in Figure 2. The most frequent reason for death was due to cancer (51.2%) followed by heart disease and stroke (22.1%). For the deaths occurring between 2005 and 2017, a similar trend was seen where mortality due to cancers accounted for 53.6% and heart disease 19.6%. The most common types of cancer were digestive tract neoplasms, which accounted for 36.0% of the cases. This group included but was not limited to stomach, intestine, colon, oesophageal, and pancreatic cancers. More participants with periodontitis at baseline died of overall causes compared to periodontally healthy participants (*n* = 39 [13.8%] vs. *n* = 106 [7.8%], *p* = 0.001). Furthermore, more periodontitis participants died of cancer (*n* = 22 [7.8%] vs. *n* = 53 [3.9%], *p* = 0.004). Surprisingly, in this respect, there was no statistically significant difference in mortality due to heart disease (*n* = 5 [1.8%] vs. *n* = 27 [2.0%], *p* = 0.812).

Medication purchases of the subjects during 2005–2017 are given in Table 2. Compared with the surviving participants, 87.6% of the deceased participants had purchased significantly more alimentary tract and metabolism medications, mainly anti-emetics and anti-nauseants, anti-diarrhoea, intestinal anti-inflammatory, or anti-infective agents. Anti-infectives, nervous system medications, and musculoskeletal system medications (mainly non-steroidal anti-inflammatory drugs and opioids) had also been frequently purchased by the participants who had died.

**Table 2:** Purchases of the main anatomical therapeutic chemical groups of medications in the cohort during the years 2005–2017.

	2005–2017	
	Alive	Dead
	(*n* = 1495)	(*n* = 97)
Median of medication purchases	85 SD: 202.2	104 SD: 314.1
Median of subgroups of medications	12 SD: 7.4	15 SD: 7.8
Alimentary tract and metabolism	1024 (68.5%)	85 (87.6%)***
Blood and blood-forming organ	700 (46.8%)	61 (62.9%)**
Cardiovascular system	937 (62.8%)	71 (73.2%)*
Dermatologicals	895 (59.9%)	63 (64.9%)
Genito urinary system and sex hormones	775 (51.8%)	40 (41.2%)*
Systemic hormonal preparations, excl. sex hormones and insulins	516 (34.5%)	60 (61.9%)***
Anti-infectives for systemic use	1287 (86.1%)	86 (88.7%)
Antineoplastic and immunomodulating agents	141 (9.4%)	32 (33.0%)***
Musculoskeletal system	1080 (72.2%)	68 (70.1%)
Nervous system	1111 (74.3%)	79 (81.4%)
Antiparasitic products, insecticides, and repellents	336 (22.5%)	17 (17.5%)
Respiratory system	1083 (72.4%)	68 (70.1%)
Sensory organs	793 (53.0%)	44 (45.4%)

SD: standard deviation.

These data are based on Chi^2^-tests where the distribution of data is on four cells but only two of those are shown. For a complete distribution of data contact the first author. For all variables, the degree of freedom (*dF*) is 1. The mean and median of medication purchases are only descriptive statistics and states the number of medication purchases of the sample. The percentages are calculated for each column.

* *P*-value < 0.05.

** *P*-value < 0.01.

*** *P*-value < 0.001.

**Figure 3:**
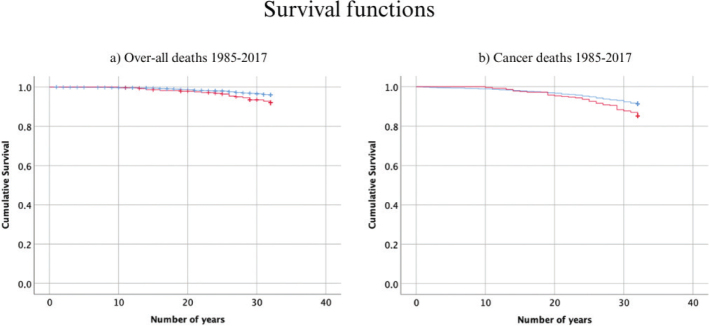
Kaplan-Meier survival curves for participants with periodontitis and periodontally healthy participants at baseline for the years 1985–2017. In red is the survival curve for participants with periodontitis at baseline and in blue the survival curve for periodontally healthy at baseline. A depicts the over-all survival curve for all medical related deaths and B depicts cancer-specific deaths.

The Kaplan-Meier analysis showed a clear difference in survival between participants with periodontitis at baseline and periodontally healthy at baseline as seen in Figure 3. The Tarone-Ware-test indicates that the difference between the curves were significant for both curve (a) over-all mortality (χ^2^(1) = 9.70, *p* = 0.002) and curve (b) cancer-specific mortality (χ^2^(1) = 8.15, *p* = 0.004).

Periodontitis at baseline was positively and significantly correlated to the mortality risk due to cancers in 1985–2017, as given in detail in Table 3. The mortality risk for all causes of death in 1985–2017 was positively and significantly correlated to loss of two or more teeth, as well as to a higher than median gingival and calculus index when adjusted for gender, tobacco use, a hospital diagnosis before 1985, and lower socioeconomic status at baseline. Furthermore, the HRs for periodontitis (HR = 1.845, confidence interval [Cl] = 1.07–3.19) were greater for deaths due to cancers than for tobacco use (HR = 1.79, Cl = 1.1–2.89); however, the difference is not significant due to the overlap in the HRs.

**Table 3:** Cox’s proportional hazards regression analysis over all-cause mortality and mortality due to cancers in the years 1985–2017.

	Model 1		Model 2	
	HR	*p*	HR	*p*
	(95% Cl)		(95% Cl)	
**All-cause mortality 1985–2017**				
Periodontitis	1.81 (1.25–2.61)	0.002	1.31 (0.87–1.97)	0.194
Loss of 2 or more teeth	1.73 (1.24–2.42)	0.001	1.47 (1.03–2.11)	0.034
PI	1.54 (1.11–2.14)	0.009	1.36 (0.98–1.96)	0.068
CI	1.81 (1.29–2.54)	<0.001	1.44 (1.00–2.07)	0.050
GI	1.87 (1.33–2.62)	<0.001	1.52 (1.06–2.19)	0.023
**Cancer mortality 1985–2017**				
Periodontitis	2.04 (1.24–3.35)	0.005	1.85 (1.07–3.19)	0.028
Loss of 2 or more teeth	1.60 (1.00–2.55)	0.051	1.42 (0.86–2.33)	0.170
PI	1.11 (0.70–1.76)	0.647	1.20 (0.72–1.98)	0.489
CI	1.59 (1.00–2.53)	0.049	1.51 (0.92–2.48)	0.103
GI	1.48 (0.94–2.35)	0.092	1.43 (0.88–2.33)	0.154

HR: Hazard ratio; CI: Confidence interval; PI: High plaque index; CI: High calculus index; GI: High modified gingival index.

The Cox’s proportional hazards regression survival analyses were adjusted for the following covariates:

Model 1: non-adjusted

Model 2: adjusted for sex, age, smoking, diagnosis before 1985 and lower socioeconomic status. Different oral health variables recorded clinically at baseline in 1985: Periodontitis at baseline. The loss of two or more teeth at baseline.

Participants who had periodontitis at baseline had a significantly higher all-cause mortality risk and particularly mortality risk due to cancers, also when adjusted for medication purchases as given in Table 4. Consequently, for participants with periodontitis at baseline a significant higher all-cause mortality (HR = 2.315, Cl = 1.459–3.672, *p* < 0.001) and cancer-specific mortality risk (HR = 3.030, Cl = 1.631–5.629, *p* <0.001) could be found even when adjusting for the high medication purchasing above the median (polypharmacy), while not including the specific types of medications. In Table 4, however, the polypharmacy as a sole risk factor is not included.

**Table 4:** Cox’s proportional hazards regression analysis over all-cause mortality and mortality due to cancers in the years 2005–2017.

	Model 1		Model 2		Model 3	
	HR	*p*	HR (Cl 95%)	*p*	HR	*p*
	(95% Cl)		(95% Cl)		(95% Cl)	
**All-cause mortality in 2005–2017**						
Periodontitis	1.93 (1.24–3.00)	0.004	2.32 (1.46–3.67)	<0.001	2.20 (1.36–3.55)	0.001
Loss of 2 or more teeth	1.39 (0.92–2.12)	0.122	1.68 (1.09–2.59)	0.019	1.74 (1.12–2.72)	0.015
PI	1.36 (0.91–2.02)	0.139	1.30 (0.84–1.98)	0.247	0.98 (0.62–1.54)	0.930
CI	1.74 (1.16–2.26)	0.008	1.78 (1.16–2.72)	0.008	1.83 (1.18–2.83)	0.007
GI	1.57 (1.05–2.36)	0.030	1.57 (1.03–2.42)	0.038	1.43 (0.92–2.21)	0.110
**Cancer mortality in 2005–2017**						
Periodontitis	2.22 (1.23–4.00)	0.008	3.03 (1.63–5.63)	<0.001	2.44 (1.28–4.68)	0.007
Loss of 2 or more teeth	1.43 (0.81–2.53)	0.219	1.69 (0.93–3.07)	0.086	2.05 (1.10–3.83)	0.024
PI	1.12 (0.64–1.94)	0.696	1.25 (0.69–2.28)	0.461	0.83 (0.43–1.58)	0.561
CI	1.56 (0.89–2.73)	0.118	1.77 (0.99–3.18)	0.054	1.86 (1.01–3.43)	0.048
GI	1.33 (0.77–2.30)	0.307	1.53 (0.86–2.74)	0.149	1.23 (0.67–2.26)	0.496

HR: Hazard ratio; CI: Confidence interval; PI: High plaque index; CI: High calculus index; GI: High modified gingival index.

The Cox’s proportional hazards regression survival analyses were adjusted for the following covariates:

Model 1: non-adjusted

Model 2: adjusted for sex, age, smoking, diagnosis before 1985, and lower socioeconomic status.

Model 3: adjusted for all covariates in model 2 and the medication purchases. The medication purchases include the main groups of medications according to the ATC. Different oral health variables recorded clinically at baseline in 1985: Periodontitis at baseline. The loss of two or more teeth at baseline.

In addition, a higher calculus index at baseline correlated to a higher all-cause mortality risk, but neither baseline tooth loss, a higher plaque index nor a higher gingival index score, respectively, appeared to increase the mortality risk. For deaths due to cancers, alimentary tract and metabolism medications (HR = 6.348, Cl = 1.379–29.231, *p* = 0.018), systemic hormones (HR = 2.591, Cl = 1.259–5.332, *p* = 0.010), and antineoplastic and immunomodulating agents (HR = 4.077, Cl = 2.105–7.895, *p* < 0.001), respectively, were positively associated with high mortality risk. Only sensory organ medications showed a significantly protective HR = 0.452 (Cl = 0.241–0.850, *p* = 0.014). Similarly, for all-cause mortality alimentary tract and metabolism medications (HR = 3,370, Cl = 1.608–7.063, *p* = 0.001) and systemic hormones (HR = 2.006, Cl = 1.230–3.272, *p* = 0.005) were positively associated with high mortality risk. In addition, for all-cause mortality, genital urinary system drugs and sex hormones (HR= 0.626, Cl = 0.400–0.982, *p* = 0.041) were negatively correlated with the mortality risk in the cohort.

## Discussion

The main finding of the current study was that periodontitis at baseline was indeed found to be a risk factor for both cancer-related and all-cause mortality among our participants during the years from 1985 to 2017. In addition, having lost two or more teeth at the age of 30–40 years, and having higher dental calculus and gingival index scores at baseline were also identified as risk factors for overall mortality. Moreover, the analysis of medication purchases revealed a pattern suggesting that medicines for gastroenterological and metabolic disorders, and systemic hormones, may be linked to increased mortality risk. Still, no association could be found between periodontitis at baseline and CVD mortality risk which was astonishing in reference to earlier literature [23].

The strengths of our present study are its long follow-up period, robust and multifactorial registers at hand, and the inclusion of all medication purchases as covariables. Inclusion of age, socioeconomic factors, hospital diagnosis before 1985, and tobacco usage at baseline, provide a possibility of eliminating bias. Research suggests that socioeconomic factors and behavioural factors can partly explain the associations between poor oral health and mortality [24]. These, too, were considered in the present study.

Yet, several limitations of this long-term register study need to be discussed. First, alcohol use, obesity and lifestyle habits except tobacco consumption could not be included in the analyses due to lack of data. Since a poor lifestyle affects systemic health, quality of life, and oral health, this lack of information is unfortunate. Secondly, because of the observational nature of the study causal conclusions cannot be drawn. Further, there were no data of changes throughout the observation in oral behaviour, periodontal treatments, and tobacco usage. Furthermore, no clinical follow-up examinations of the whole cohort after 1985 could be performed, which is another limitation of the study and should be taken into consideration when interpreting the results. In addition, it should be pointed out that in 1985 when this study started, there was no consensus on the diagnosis criteria of periodontitis. The indexes currently in use had not been developed at that time.

Periodontitis has been associated with several cancer types [25–28]. This aligns with the findings of our study. Possible theories include dysbiosis, chronic inflammation, immune evasion, and direct (epi)genetic damage to the epithelial cells caused by periodontal pathobionts and their toxins [29]. Periodontal pathogens have been detected in several cancers, including, but not limited to, oral and gastrointestinal cancers [30], and in aerodigestive tumour tissue in general [31]. We have observed earlier in our research group that in this cohort there was an association between breast cancer incidence, periodontal disease, and missing molars [32]. Research regarding the effect of periodontitis on cancer immunotherapy is ongoing but there is a clear need for further study [33].

Our findings further confirm results from previous studies that have linked periodontitis or poor oral health, including tooth loss, with increased all-cause mortality risk. For instance, both objective and subjective poor oral health increases the risk for overall mortality among older adults in the US [34]. In the same study, severe periodontitis was significantly associated with a higher risk of overall mortality according to Cox’s proportional hazards regression. In another study, a hospital diagnosis of periodontitis significantly increased the risk of comorbidities, CVD, and all-cause mortality [35]. In addition, self-rated poor oral health has been linked to an increased risk of CVD, diabetes, osteoporosis, and all-cause mortality among women [36]. Self-reported poor oral health and edentulism have also been associated with an increased risk for overall mortality in older, previously healthy adults [37, 38].

In another cohort study from the Stockholm region, poor oral health was found to increase the risk of mortality due to CVD and cancers and, indeed, linked to overall mortality [14]. In that study, contrary to our present investigation, an oral health score was constructed in the analyses that contained several variables including the number of lost teeth, marginal bone loss, number of teeth with caries and apical lesions, dental plaque index, and Russel’s periodontal index. Although in that study, a health score was used, while we used a multitude of different oral health variables, the findings do align. The confounding factors included age, sex, smoking, and social group, which were the same as in our current study [14].

In register studies and regression models, controlling for confounding factors is important. In our present study, we controlled for age, sex, tobacco usage, low socioeconomic status, and the existence of earlier hospital diagnoses. Furthermore, we included the number of medication purchases between the years 2005 and 2017 to reflect the comorbidity burden of the participants. Analysing the different groups of medications purchased thus gave a picture of the underlying diseases, that is, why the medications had been prescribed in the first hand.

The association of periodontitis and systemic diseases including CVD has been explored in several earlier studies [2, 4, 39]. Furthermore, many studies suggest a relationship between tooth loss and an increased risk of CVD hospitalisation and overall mortality, and CVD mortality specifically [23, 40]. Interestingly, however, in the current study, we did not see an association between periodontitis and CVD mortality. Yet, the participants who had been diagnosed with periodontitis at baseline did purchase more CVD medications later in life as we have reported [41]. Earlier our research group has also found that periodontal disease did associate with early atherosclerotic carotid lesion development [42]. Nevertheless, the association between periodontitis and CVD-related death could not be found in our present investigation. This may be due to the purchases of CVD medications later in life by the participants. These medications are obviously used to treat CVD and may thus decrease or postpone respective CVD-caused mortality. However, CVD medication can cause hyposalivation [43]. Hence, medication used by the participants might also have affected their oral health later in life. Although in the present investigation we had more than 35-year-long observation time, we could only include the medication purchases for the last 12 years because of the construction of the national pharmacology register that was available for the study in Sweden.

Finally, our findings also showed that in the deaths due to cancer, gastrointestinal drugs and medications for metabolic diseases, systemic hormones, and antineoplastic and immunomodulating agents, were all associated with an increased mortality risk. Since polypharmacy is on the rise, the medication purchases of the participants are important to keep in mind in the clinical setting. For example, gastrointestinal medications include anti-nausea drugs and are often a necessary part of cancer treatment and medication treatment as such medications prolong life and cure or ameliorate the disease. However, polypharmacy has also earlier been associated with an increased risk of mortality [44]. In a recent meta-analysis, pharmacological counselling and intervention have been shown to reduce the number of medications used by the participants, and lower clinical readmissions within 30 days. However, in that study, a significant effect on all-cause mortality could not be found [45]. Other researchers have suggested that in cancers with a typically favourable prognosis, polypharmacy is associated with a higher risk of mortality, too [46]. As said, in our present study there is a gap in the medication records between baseline and the year 2005. Since medication purchases are tied to medical conditions and these may vary within and in-between the years, this lack of data is an evident weakness.

Deterioration in oral function can be seen in deterioration in general health, and oral frailty increases the risk of physical frailty, sarcopenia, and mortality, particularly of the elderly [47]. This study found that periodontitis diagnosed earlier in life was found to be a risk factor for both overall and cancer-related mortality. However, further research is still needed to understand the exact pathways that explain how chronic low-grade inflammation, such as in periodontitis, influences systemic health and affects survival. Thus, to confirm the findings of our study, further investigations are needed with more diverse populations than the fairly homogenous Caucasian Swedish population investigated here. The role of polypharmacy on oral health and in general in the perspective of this study area also calls for more investigations.

## Declarations

### Data availability statement

Data may be obtained by request from the first author.
